# Critical Role of Plasmacytoid Dendritic Cells in Regulating Gene Expression and Innate Immune Responses to Human Rhinovirus-16

**DOI:** 10.3389/fimmu.2017.01351

**Published:** 2017-10-25

**Authors:** Yang Xi, Niamh M. Troy, Denise Anderson, Olga M. Pena, Jason P. Lynch, Simon Phipps, Anthony Bosco, John W. Upham

**Affiliations:** ^1^Lung and Allergy Research Center, Diamantina Institute, The University of Queensland, Brisbane, QLD, Australia; ^2^Systems Immunology, Telethon Kids Institute, The University of Western Australia, Perth, WA, Australia; ^3^Respiratory Immunology Group, QIMR Berghofer Medical Research Institute, Herston, QLD, Australia; ^4^Department of Respiratory Medicine, Princess Alexandra Hospital, Woolloongabba, QLD, Australia

**Keywords:** plasmacytoid dendritic cells, human rhinovirus, human rhinovirus responsive genes, plasmacytoid dendritic cell-dependent gene expression, innate immune response

## Abstract

Though human rhinoviruses (HRVs) are usually innocuous viruses, they can trigger serious consequences in certain individuals, especially in the setting of impaired interferon (IFN) synthesis. Plasmacytoid dendritic cells (pDCs) are key IFN producing cells, though we know little about the role of pDC in HRV-induced immune responses. Herein, we used gene expression microarrays to examine HRV-activated peripheral blood mononuclear cells (PBMCs) from healthy people, in combination with pDC depletion, to assess whether observed gene expression patterns were pDC dependent. As expected, pDC depletion led to a major reduction in IFN-α release. This was associated with profound differences in gene expression between intact PBMC and pDC-depleted PBMC, and major changes in upstream regulators: 70–80% of the HRV activated genes appeared to be pDC dependent. Real-time PCR confirmed key changes in gene expression, in which the following selected genes were shown to be highly pDC dependent: the transcription factor *IRF7*, both *IL-27* chains (*IL-27p28* and *EBI3*), the alpha chain of the IL-15 receptor (*IL-15RA*) and the IFN-related gene *IFI27*. HRV-induced IL-6, IFN-γ, and IL-27 protein synthesis were also highly pDC dependent. Supplementing pDC-depleted cultures with recombinant IL-15, IFN-γ, IL-27, or IL-6 was able to restore the IFN-α response, thereby compensating for the absence of pDC. Though pDC comprise only a minority population of migratory leukocytes, our findings highlight the profound extent to which these cells contribute to the immune response to HRV.

## Introduction

Human rhinoviruses (HRVs) are ubiquitous single stranded RNA viruses that are responsible for most common cold cases. In healthy people, HRV infections generally cause relatively minor symptoms and are largely of nuisance value. However, this innocuous virus can have serious consequences in immunosuppressed individuals. HRV infections have also been linked to the onset of asthma in young children and to severe exacerbations of established asthma and chronic obstructive pulmonary disease (1–3). There is thus considerable interest in understanding the pathogenesis of HRV infections and the factors that mediate host protection against this virus.

Type I interferons (IFN-Is), including the various IFN-α subtypes and IFN-β, are important mediators of host protection against virus infections. Lack of IFN-I production can lead to reduced antiviral responses enabling higher viral loads and increased disease severity. Studies have shown that mice deficient in IFN-I production are more susceptible to many viral infections such as vesicular stomatitis virus, Semliki forest virus, vaccinia virus, and lymphocytic choriomeningitis virus than wild-type mice (4). In human asthma and murine models, HRV infection in the setting of low IFN-I production is closely linked to severe, typically type 2, airway inflammation and increased viral replication, features of disease that are thought to predispose to acute asthma exacerbations (5, 6). HRV induces transcription of numerous genes in the airway mucosa, many of which are IFN responsive genes or closely associated with IFN synthesis (7, 8). *In vitro* studies indicate that HRVs induce a variety of cell types to synthesize IFNs and other cytokines, including lung structural cells such as airway epithelial cells (9) and fibroblasts (10), and bone marrow-derived cells such as alveolar macrophages (11) and plasmacytoid dendritic cells (pDCs) (12).

Human pDC are characterized as Lin^−^MHC^−^II^+^CD303 (BDCA2)^+^CD304 (BDCA4)^+^ cells (13) and play a key role in IFN-I production during virus infections (14). Although a relatively rare cell type [~0.4% of total peripheral blood mononuclear cells (PBMCs)], pDC dedicate much of their transcriptome to IFN synthesis and are prearmed with virus-sensing pattern recognition receptors such as toll-like receptors (TLR7 and TLR9). pDC are able to produce 100–1,000 times more IFN-I than any other cell following exposure to DNA and RNA viruses, and thus are regarded as “natural IFN-I producers” (15). This has been attributed to the ability to rapidly activate interferon regulatory factor 7 (IRF7), a master regulator of IFN-I expression (16). This IRF7 signaling pathway within pDC appears especially important for rapidly inducing IFN-I. However, there are considerable knowledge gaps around how pDC regulate HRV-induced immune responses. Transgenic mouse models have been designed to allow conditional depletion of pDC during virus infections, demonstrating the important role of pDC in mediating early antiviral IFN responses (17). However, confirming these findings in humans presents considerable challenges.

Our group recently developed a system allowing pDC depletion from cultured human PBMC as an indirect but powerful method to better understand human pDC function. This study demonstrated that pDC constrain type 2 responses to HRVs, an important regulatory mechanism that may be deficient in asthma and other allergic disorders (14, 18). To better understand the regulatory properties of human pDC, we therefore undertook a holistic approach using gene expression microarray analysis and pDC depletion in order to (i) determine the effects of HRV on gene expression patterns in PBMC from healthy people and (ii) establish the extent to which these gene expression patterns are dependent on pDC.

## Materials and Methods

### Study Cohorts

The project recruited eighteen healthy adult volunteers (mean age 35 ± 9 years). All subjects answered a questionnaire detailing symptoms of respiratory disease and underwent skin prick testing (SPT) against a panel of nine common inhaled allergens (*Aspergillus fumigatus*, Alternaria, Bahia, Ryegrass, Johnson, Bermuda, house dust mite, cat, and dog dander). All participants had no history of allergic disease or respiratory disease and a negative response to SPT. They had no family history of atopic disease or lung disease and were not taking any supplements or medications at the time when the blood was taken. The Metro South Human Research Ethics Committee approved the study, and all subjects provided written informed consent.

### Rhinovirus Generation and Titration

Human rhinoviruses strain 16 (RV16) stocks were generated by passage in Ohio HeLa cells, as described previously (19) followed by purification over an OptiPrep gradient (Sigma-Aldrich). To define the optimal concentration of RV16, the 50% tissue culture-infective dose (TCID50) was determined as previously described (18), and all cell stimulations used a multiplicity of infection of 1 (MOI = 1).

### PBMC Separation and Depletion of Peripheral pDC

Peripheral blood mononuclear cells were isolated from human fresh whole blood by density gradient centrifugation. PBMC were depleted of pDC using CD303 (BDCA-2) immunomagnetic beads with an AutoMACs apparatus according to the manufacturer’s instructions (Miltenyi Biotec, Germany). In selected experiments, pDC were also depleted by cell sorting with an Astrios Sorter as per manufacturer’s instructions (BD Bioscience) in the Translational Research Institute flow cytometry core facility. Purity of pDC depletions were assessed using flow cytometry as described previously (18).

### Cell Culture and Preparation

Peripheral blood mononuclear cells or pDC-depleted PBMC were cultured at 2 × 10^6^ cell/ml in media (RPMI 1640 supplemented with 2% heat-inactivated fetal bovine serum (HI-FBS), penicillin, streptomycin, and glutamine) as previously described (20). Briefly, 250 μl/well cell resuspension was cultured in a 96-well U-bottom plate, with 8 replicates in each group. The cells were rested at 37°C with 5% CO_2_ and 95% humidity overnight and then subsequently stimulated with RV16 or left unstimulated, and further incubated for 24 h. Following culture, plates were centrifuged at 750 × *g* for 5 min and the supernatants were pooled and harvested for cytokine quantification by enzyme-linked immunosorbent assay (ELISA). The cell pellets were pooled and resuspended in 200 µl of RNA protect, and stored in −80°C for RNA extraction using the RNeasy mini kit (QIAGEN, Australia).

### Microarray-Based Expression Profiling Studies

Total RNA from PBMC samples preserved in RNAprotect was extracted by RNeasy mini Kit together with RNase free DNase set (QIAGEN) according to manufacturer’s instructions. The quantity and quality of the RNA sample was determined using the Agilent RNA6000 Nano kit on the 2100 Bioanalyzer (Agilent, Amstelveen, The Netherlands). Samples with RNA integrity number >7 were selected for further analysis. Total RNA samples (*n* = 15) were biotinylated and amplified using the Ilumina^®^ TotalPrep™ RNA Amplification Kit (Ambion, Austin, TX, USA) as per manufacturer’s instructions with a standardized input amount of 500 ng. Whole-genome transcriptional profiling was performed using Illumina Human HT-12 microarrays (SanDiego, CA, USA) as per manufacturer’s instructions. These were performed by the microarray facility at the Diamantina Institute, University of Queensland. The microarray data were processed and analyzed at Telethon Kids Institute (University of Western Australia, Perth). The raw data are available from Gene Expression Omnibus repository (accession number GSE99858).

### Gene Microarray Data Analysis

Illumina BeadStudio summary probe and summary control probe profiles were read into R (21) using the lumiR.batch() function available in the *lumi* package (22), and using the read.ilmn() function available in the *limma* package (23). Both *lumi* and *limma* were used to read in the data as each package offers different quality control checks. The proportion of expressed microarray probes for each sample was estimated using the propexpr() function available in the *limma* package, and this check did not reveal any outlying arrays. Background correction and normalization was performed using the neqc() function (24) available in *limma*, which uses the normal-exponential convolution model for background correction followed by quantile normalization. Distribution of probes for each array was assessed using boxplots and density plots, before and after normalization, and no outlying arrays were identified. The *lumi* package, plotSampleRelation() function, was used to assess sample similarity through multidimensional scaling, and no outlying samples were identified.

12,847 poor quality probes were removed based on annotation from Barbosa-Morais et al. (25). A further 6,310 non-responding probes were removed based on Illumina’s detection *p*-values. The *illuminaHumanv4.db* package (26) was used to annotate probes with gene symbols and 4,532 probes without gene symbols were removed. 44 samples and 23,634 probes were included in the differential expression analysis. The *limma* package was used for differential expression analysis to test for rhinovirus responsive genes in PBMCs, pDC-depleted PBMCs and for differences in rhinovirus responsive genes in pDC-depleted PBMCs versus PBMCs. Array quality weights were calculated using the arrayWeights() function, and these weights were used in the linear model as a measure of array reliability (27). Correlation between samples taken from the same individual was adjusted for through use of the duplicateCorrelation() function (28). *p*-Values were adjusted for multiple testing using the Benjamini and Hochberg method (29). Three-dimensional principal component analysis (PCA) plots were generated using the *rgl* package (30) and heat maps were produced using the heatmap.2() function of the *gplots* package (31).

### Upstream Regulator Analysis

Differentially expressed genes (adjusted *p*-value < 0.01) were interrogated with Upstream Regulator Analysis (Ingenuity Systems, Redwood City, CA, USA) to identify putative molecular drivers of the observed expression patterns (32, 33). This analysis leverages experimentally derived cause-and-effect molecular relationships extracted from the literature. Two statistical measures are calculated: (i) the overlap *p*-value is based on enrichment of known target genes for each upstream regulator amongst the list of differentially expressed genes and (ii) the activation *Z*-score measures the pattern match between the direction of the observed gene expression changes (up-/downregulation) and the predicted pattern based on prior experimental evidence. An absolute activation *Z*-score greater than 2 was deemed statistically significant (32).

### Real-time PCR (RT-PCR) Validation Studies

Extracted RNA (500–800 ng per sample) was reverse transcribed using SensiFAST cDNA Synthesis Kit (BIOLINE), according to the manufacturer’s instructions. Gene expression was assessed by RT-PCR by LightCycler 480 (Roche Applied Science) with SensiFAST SYBR No-ROX kit (Bioline). *UBE2D2* that has been previously assessed to be stably expressed in PBMC with/without stimulation of RV16 (34) and *B2M* were used as another reference gene for normalization of the RT-PCR data. Table S1 in Supplementary Material shows the primers sequence used to amplify *CD303, IRF7, IL-27p28, EBI3, IL-15RA, IFI27, IL-12p35, UBE2D2*, and *B2M*. The data was analyzed using the Pfaffl method and the results are expressed as a ratio of stimulated to control (unstimulated) samples, with a fold change of 1.0 representing unstimulated expression levels.

### “Rescue Experiments” Using pDC-Depleted Culture Supplemented with Recombinant Cytokines

Intact PBMC or pDC-depleted PBMC were preincubated with recombinant human IL-27, IFN-γ, IL-6, IL-15, or IFN-β at 10 ng/ml for 1 or 4 h. Both cell populations were then stimulated with RV16 and further incubated for up to 24 h.

### ELISA Assays

Cytokines were measured in culture supernatants by ELISA according to the manufacturer’s instructions. IL-6 and IFN-γ assays used commercially available paired antibodies and recombinant cytokines (BD Biosciences, Franklin Lakes, NJ, USA; limit of detection = 3.91 pg/ml for both cytokines). IL-27 (R&D systems) was assayed *via* commercial ELISA kit (limit of detection = 19.53 pg/ml). IFN-α was measured by VeriKine™ Human IFN-α ELISA kit that detects multiple IFN-α subtypes (PBL assay Science; limit of detection = 12.5 pg/ml).

### Intracellular Cytokine Staining

Intracellular cytokine staining was used to assess the extent to which IFN-α was produced by different populations, including pDC, myeloid dendritic cells (mDCs), and monocytes, 24 h post-RV16 stimulation. PBMCs (1 × 10^6^ cells/well) were seeded in a 96-U-bottom plate and stimulated with or without RV16 at 37°C with 5% CO_2_ for 18 h, and further incubated with Brefeldin A (BFA) (eBioscience) for 4 h. Cells were washed with FACs buffer (1% HI-FBS in PBS) (FBS; Bovogen biological, Australia) and incubated with normal goat IgG (Sigma Aldrich, USA) at 4°C for 15 min to block non-specific Fc binding. The cells were then surface stained with CD303-PE, CD14-PerCP, and CD1c-FITC (Miltenyi Biotec Australia) for 30 min at 4°C, then fixed and permeabilized prior to APC conjugated anti-IFN-α (Miltenyi Biotec Australia) intracellular staining for 30 min at 4°C. The cells were then washed twice with the FACs buffer, and finally fixed in 0.5% paraformaldehyde prior to analysis. Approximately 200,000 gated events per sample were collected using LSRFortessaX-20 (BD-Biosciences, USA), and the results were analyzed using the FlowJo Tree Star software (version 7.6.1). Unstimulated background values were subtracted from the data.

### Pure pDC Culture and Gene Expression Examination

In some experiments, purified pDC (*n* = 3) isolated from healthy donors by cell sorting were kindly provided by A/Prof Kristen Radford (Mater Research, University of Queensland, Translation Research Institute). Pure pDC (5 × 10^4^ cells/well) were cultured in media supplemented with 10 ng/ml of IL-3 in the presence of absence of RV16 for 24 h.

### Statistics

Statistical analysis was performed using GraphPad Prism 6 for Windows (GraphPad Software, San Diego, CA, USA) using Friedman tests with Dunn’s posttests to compare paired samples, whereas Mann–Whitney test was used to compared data from pDC-depleted PBMC and intact PBMC samples. Raw data are presented as mean ± SD. The *p*-values < 0.05 were considered significant.

## Results

### pDC Depletion Results in a Defective IFN-I Immune Response to RV16

**Figure 1:**
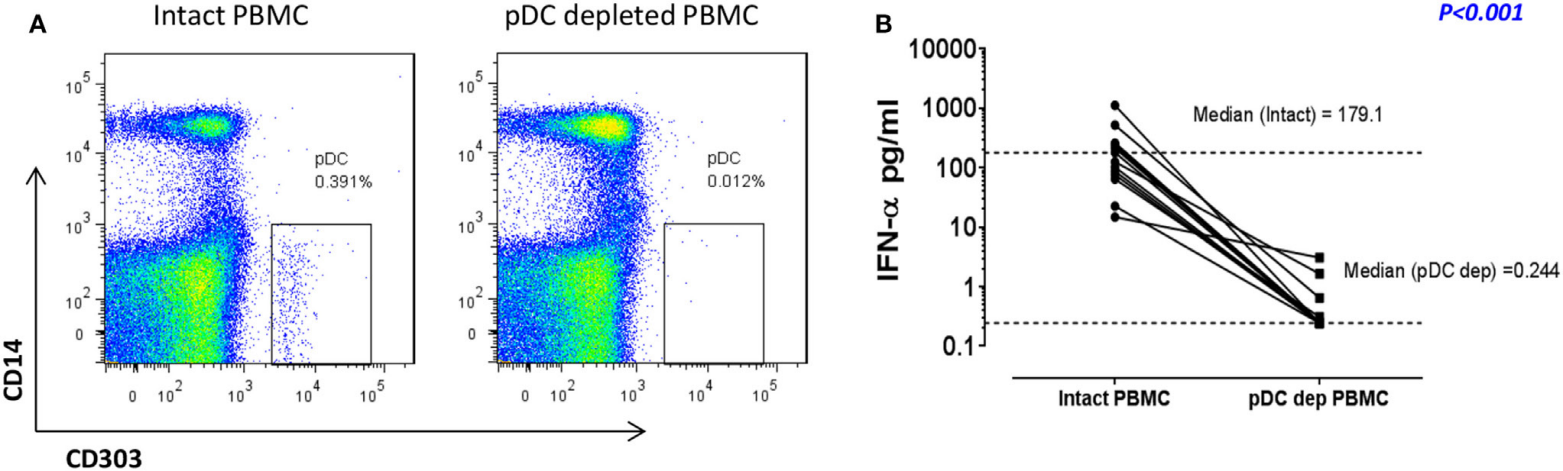
In response to human rhinoviruses strain 16 (RV16) stimulation, plasmacytoid dendritic cell (pDC)-depleted peripheral blood mononuclear cells (PBMCs) produce significantly less interferon (IFN)-α than intact PBMC. PBMC were either depleted of pDC using CD303 immunomagnetic beads or left intact. Efficiency of pDC depletion was examined with flow cytometry **(A)**. PBMC (5 × 10^5^ cells/well) were then cultured in the presence/absence of RV16 (MOI = 1) for 24 h at 37°C. IFN-α production in culture supernatant was measured by enzyme-linked immunosorbent assay (ELISA). Data shown are for the net RV16 simulated IFN-α concentration following subtraction of the IFN-α concentration in the absence of RV16 (media control). The dotted lines represent the median of each group (*n* = 15). IFN-α concentrations in pDC-depleted PBMC were significantly lower than intact PBMC (*p* < 0.001) **(B)**.

We have previously shown that pDC are responsible for more than 90% of the IFN-α production observed in RV16-stimulated human PBMC (12, 18). In the current project, we confirmed that immunomagnetic beads were able to efficiently deplete the pDC population (CD303^+^CD14^−^), with median depletion efficiency of 93% (*n* = 10). A representative flow cytometry plot is shown in Figure 1A. This was associated with a profound reduction in median IFN-α production at 24 h from 179.1 to 0.244 pg/ml (*p* < 0.001; Figure 1B). This pDC depletion system therefore provides an ideal means to assess which HRV-activated genes and gene expression pathways are dependent on pDC and/or type I IFN.

### pDC Have a Major Impact on RV16-Activated Genes and Putative Molecular Drivers

**Figure 2:**
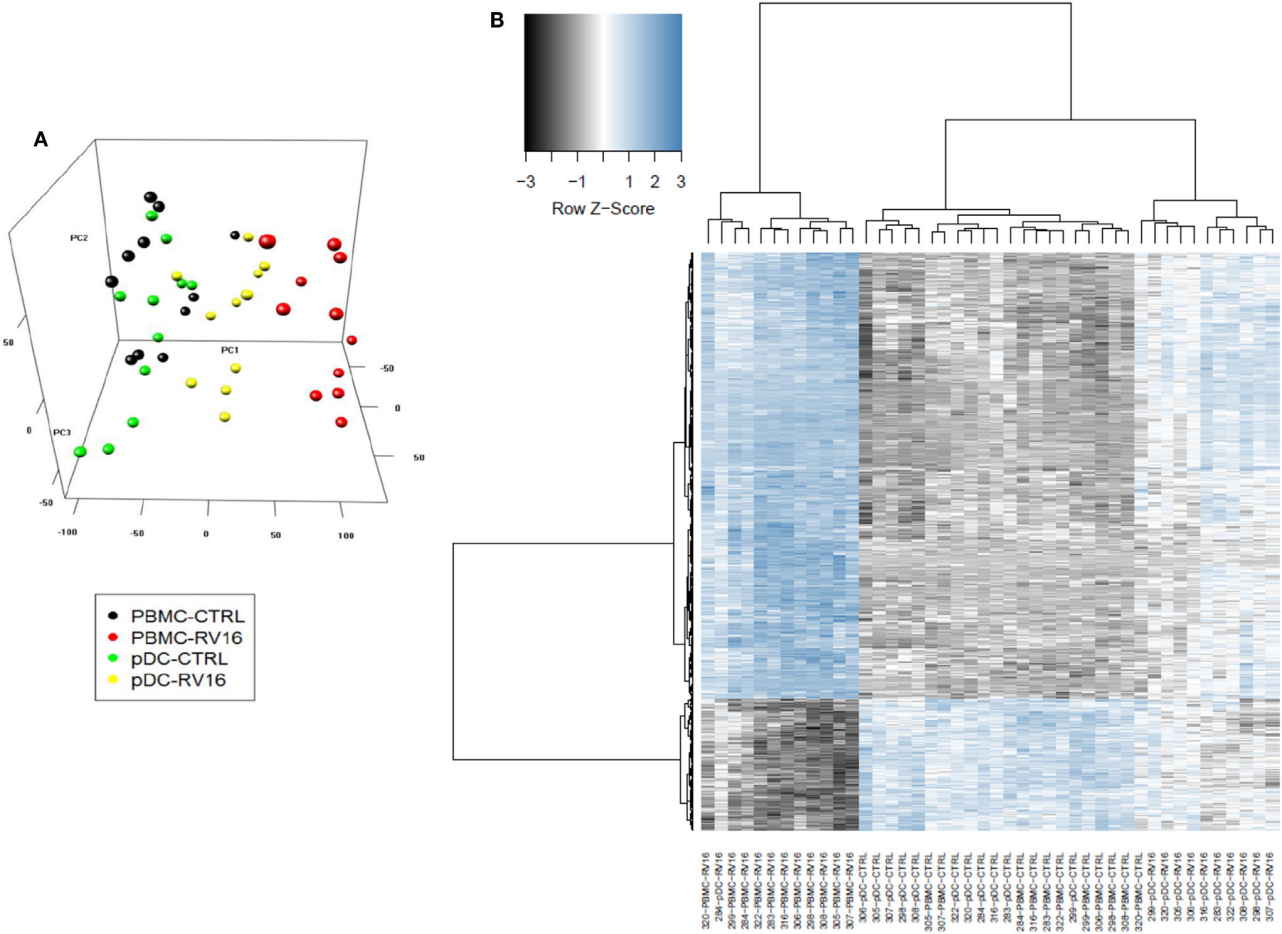
In the presence of human rhinoviruses strain 16 (RV16), intact peripheral blood mononuclear cell (PBMC) and plasmacytoid dendritic cell (pDC)-depleted PBMCs exhibit distinct patterns of gene expression. Intact PBMC and pDC-depleted PBMC (*n* = 12) were cultured with/without RV16 for 24 h. Whole-genome transcriptional profiling was performed using Illumina Human HT-12 microarray. Hierarchical cluster analysis was employed to cluster genes and samples based on the similarity of their expression patterns. RV16 response patterns in PBMC and pDC-depleted PBMC from healthy individuals (*n* = 11) were analyzed by principal component analysis. RV16-stimulated PBMC are shown in red, unstimulated PBMC are black circles. RV16-stimulated pDC-depleted PBMC are shown in yellow, and unstimulated pDC-depleted PBMC are green circles **(A)**. The heatmap illustrates the distinct difference in the RV16 induced gene expression in PBMC and pDC-depleted PBMC **(B)**.

**Figure 3:**
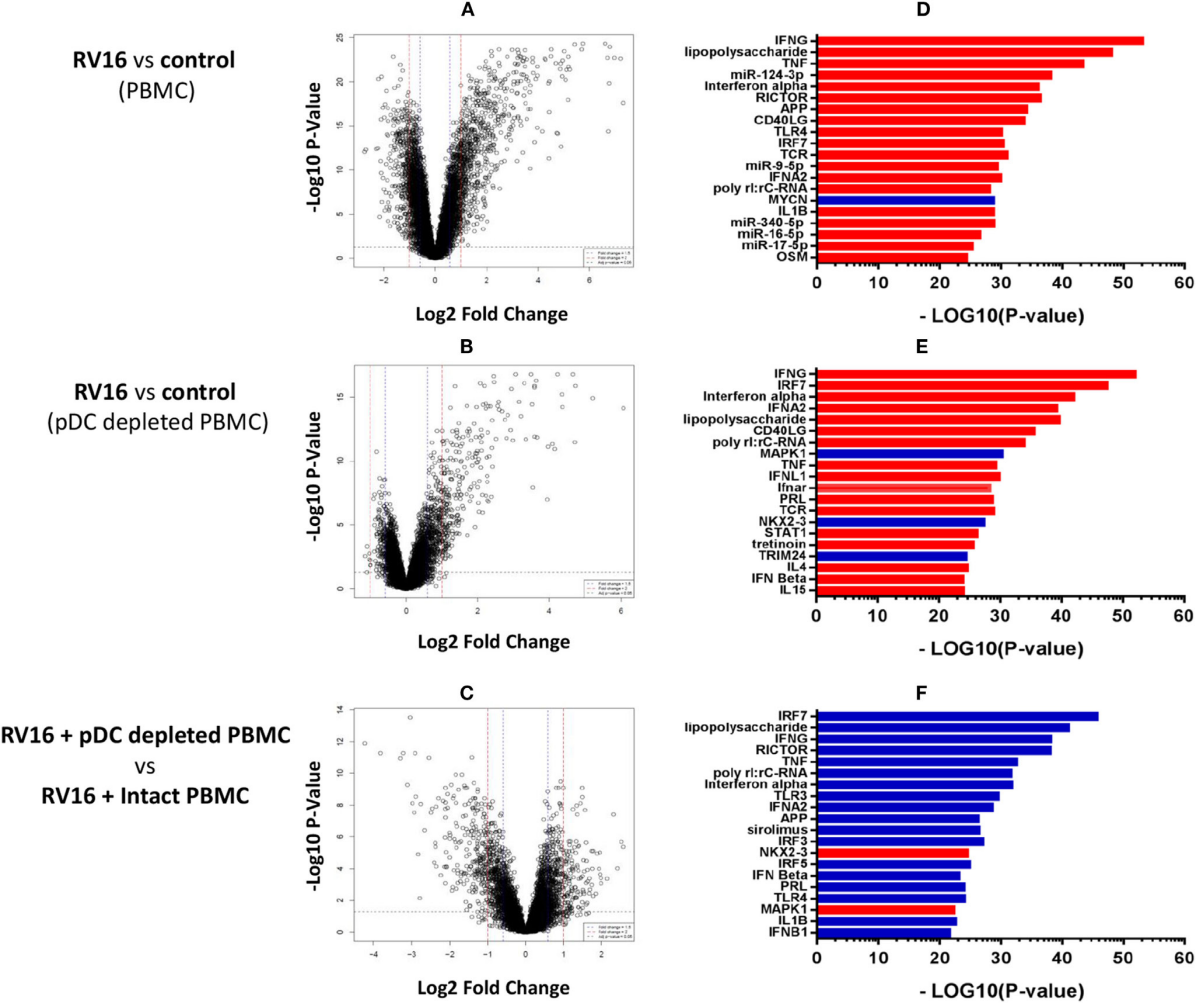
Molecular drivers of human rhinoviruses strain 16 (RV16) induced gene expression. Differentially expressed genes/molecular drivers were identified in peripheral blood mononuclear cell (PBMC) **(A,D)** and plasmacytoid dendritic cell (pDC)-depleted PBMC **(B,E)** in the presence of RV16. Comparison of RV16-stimulated pDC-depleted PBMC and intact PBMC **(C,F)**. Data analysis was performed by *limma* **(A–C)** and upstream regulator analysis **(D–F)**. The dashed horizontal line in **(A–C)** indicates FDR < 0.05. Drivers in red are predicted to be activated and those in blue are inhibited.

Intact PBMC and pDC-depleted PBMC were cultured in the presence or absence of RV16, and gene expression patterns were profiled on microarrays. PCA was employed to provide a global view of the data, and this analysis revealed that the RV16-induced intact PBMC samples clustered separately from RV16-induced pDC-depleted PBMC samples, and also from the unstimulated controls (Figure 2A). A heatmap of differentially expressed genes is illustrated in Figure 2B. The data showed that the responses were more consistent and intense in intact PBMC. Differential expression analyzes demonstrated that RV16 induced 833 differentially expressed genes in intact PBMC (Figure 3A), and 172 differentially expressed genes in pDC-depleted PBMC (Figure 3B). A direct comparison of the respective responses revealed 381 differentially expressed genes (Figure 3C).

We next employed upstream regulator analysis to identify the putative molecular drivers of the observed differential gene expression patterns (33). Two statistical measures are calculated in this analysis; the *overlap p-value* assesses target gene enrichment amongst the list of differentially expressed genes, whereas the *activation Z-score* measures the degree to which observed gene expression patterns match predicted gene expression patterns based on current knowledge. An activation *Z*-score of >2 is statistically significant (32). As illustrated in Figure 3D, the most significant candidate drivers of the HRV-stimulated responses in intact PBMC were IFN-γ, TNF, miR-124-3p, IFN-α, and RICTOR. This analysis also suggested that MYCN signaling was downregulated by HRV stimulation. A similar but distinct pattern was observed in pDC-depleted PBMC (Figure 3E): IFN-γ, IRF7, IFN-α, and IFN-α2 were again found to be the significant drivers of the HRV-stimulated responses, though in this instance MAPK1, NKX2-3, and TRIM24 signaling were also downregulated. Figure 3F highlights the major differences in HRV-stimulated responses observed in pDC-depleted versus intact PBMC. Notably, the activation of many HRV-induced pathways such as IRF7, LPS, IFN-γ, RICTOR, TNF, and IFN-α were deficient in the absence of pDC.

### What Proportion of HRV Responsive Genes Are Highly Dependent on pDC and IFN Regulated?

**Figure 4:**
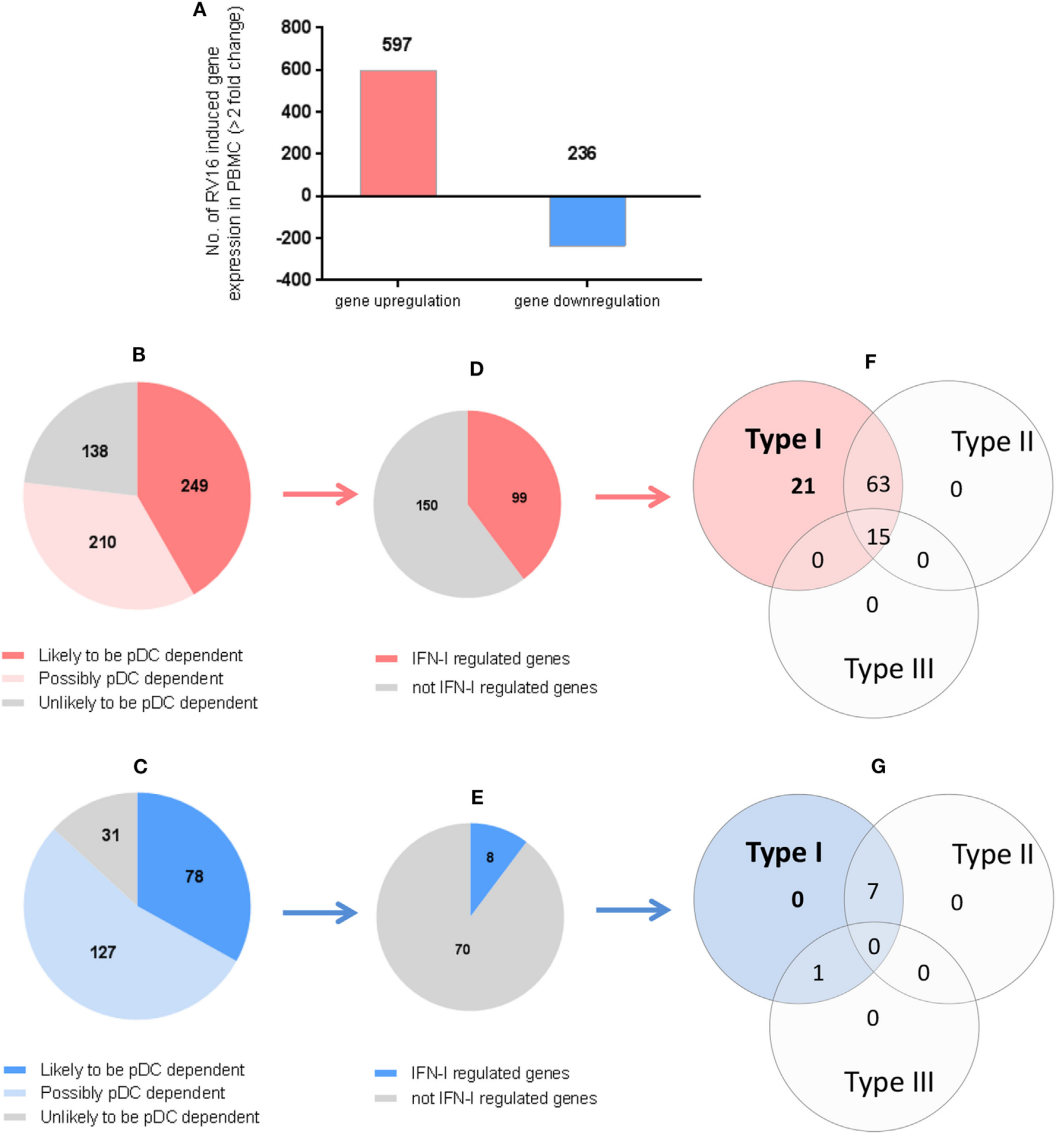
Which human rhinoviruses strain 16 (RV16) responsive genes are dependent on Plasmacytoid dendritic cell (pDC) and regulated by interferon (IFN)-I? RV16 responsive genes showing >2-fold changes have been analyzed **(A)**. Out of the total genes that are upregulated (Red) or downregulated (Blue), the numbers of pDC-dependent gene expressions have been analyzed by comparing pDC-depleted PBMC with intact PBMC. Genes that show >2-fold change are regarded as “likely to be pDC dependent”; between 1.5- and 2.0-fold changes are regarded as “possibly pDC dependent”; and <1.5-fold change and regarded as “unlikely to be pDC dependent” Numbers of genes are indicated on the pie charts **(B,C)**. Out of the total genes that are likely to be pDC-dependent expression (>2-fold), the proportion of IFN-I-regulated gene expression has been identified using *Interferome v2.01* database: **(D,E)**. Venn diagrams show the extent to which IFN-I-regulated genes are regulated solely by IFN-I, or also regulated by multiple IFN subtypes **(F,G)**.

In the intact PBMC, RV16 stimulation lead to >2-fold upregulation of 597 genes and >2-fold downregulation of 236 genes (Figure 4A). We then asked what effect pDC depletion had on these HRV responsive genes. Of the 597 upregulated genes, 249 genes showed a >2-fold change in the absence of pDC and were arbitrarily regarded as highly pDC dependent, 210 genes showed a modest change in the absence of pDC and were regarded as possibly pDC dependent, while 138 genes were minimally effected by the absence of pDC (<1.5-fold change) and were regarded as unlikely to be pDC dependent (Figure 4B).

Of the 236 downregulated genes, 78 genes showed a >2-fold change in the absence of pDC and were arbitrarily regarded as highly pDC dependent; 127 genes showed a modest change in the absence of pDC and were regarded as possibly pDC dependent, while 31 genes were minimally effected by the absence of pDC and were regarded as unlikely to be pDC dependent (Figure 4C).

We then compared our data against *Interferome V2.01*, a publicly available database of type I, II, and III IFN (IFN-II and IFN-III)-regulated genes (35) in order to infer which of the HRV-responsive and pDC-dependent genes were likely to be IFN-I regulated. Of the 249 HRV-responsive, pDC dependent, upregulated genes that are also pDC dependent (>2-fold), 99 were regarded as IFN-I-regulated genes (Figure 4D), comprising 21 genes which were solely IFN-I regulated, 63 which were IFN-I and IFN-II dual-regulated genes, and 15 which were regulated by IFN-I, IFN-II, and IFN-III (Figure 4F). Of the 78 HRV-responsive, pDC-dependent, downregulated genes, 8 were regarded as IFN-I-regulated genes (Figure 4E), comprising 0 genes which were solely IFN-I regulated, 7 which were IFN-I/II dual-regulated genes, and none of them were regulated by IFN-I, IFN-II, and IFN-III (Figure 4G). None of these HRV-responsive genes appeared to be solely IFN-II regulated or solely IFN-III regulated (Figures 4F,G). Thus, while most of the HRV activated upregulated genes are closely linked to pDC and IFN signaling, this is less apparent in the downregulated genes in which IFN signaling appears less prominent.

### Validation Experiments

Based on the microarray data and bioinformatics analyzes, HRV-responsive genes were selected for further validation by PCR, focusing on genes whose expression differed markedly between intact PBMC and pDC-depleted PBMC, or alternatively on genes predicted to be important upstream regulators. *IRF7* is regarded as a master regulator of IFN-I (16) while *IL-27p28, IL-15RA*, and *IFI27* were selected as they were among the most differentially expressed genes (Tables S2A, B in Supplementary Material). IL-27 is a heterodimeric molecule composed of *IL-27p28* and Epstein-Barr virus-induced gene 3 (*EBI3*) subunits, with both proinflammatory and anti-inflammatory functions (36). Therefore, we have additionally examined *EBI3* (also known as *IL-27B*) as it is the active part of the IL-27 heterodimer, as well as *IL-12p35*, which was included as a negative control (i.e., a gene whose expression was not predicted to be pDC dependent). Moreover, the C type lectin *CD303* (*CLEC4C*), a pDC-specific surface protein, was included to confirm the efficiency of pDC depletion.

**Figure 5:**
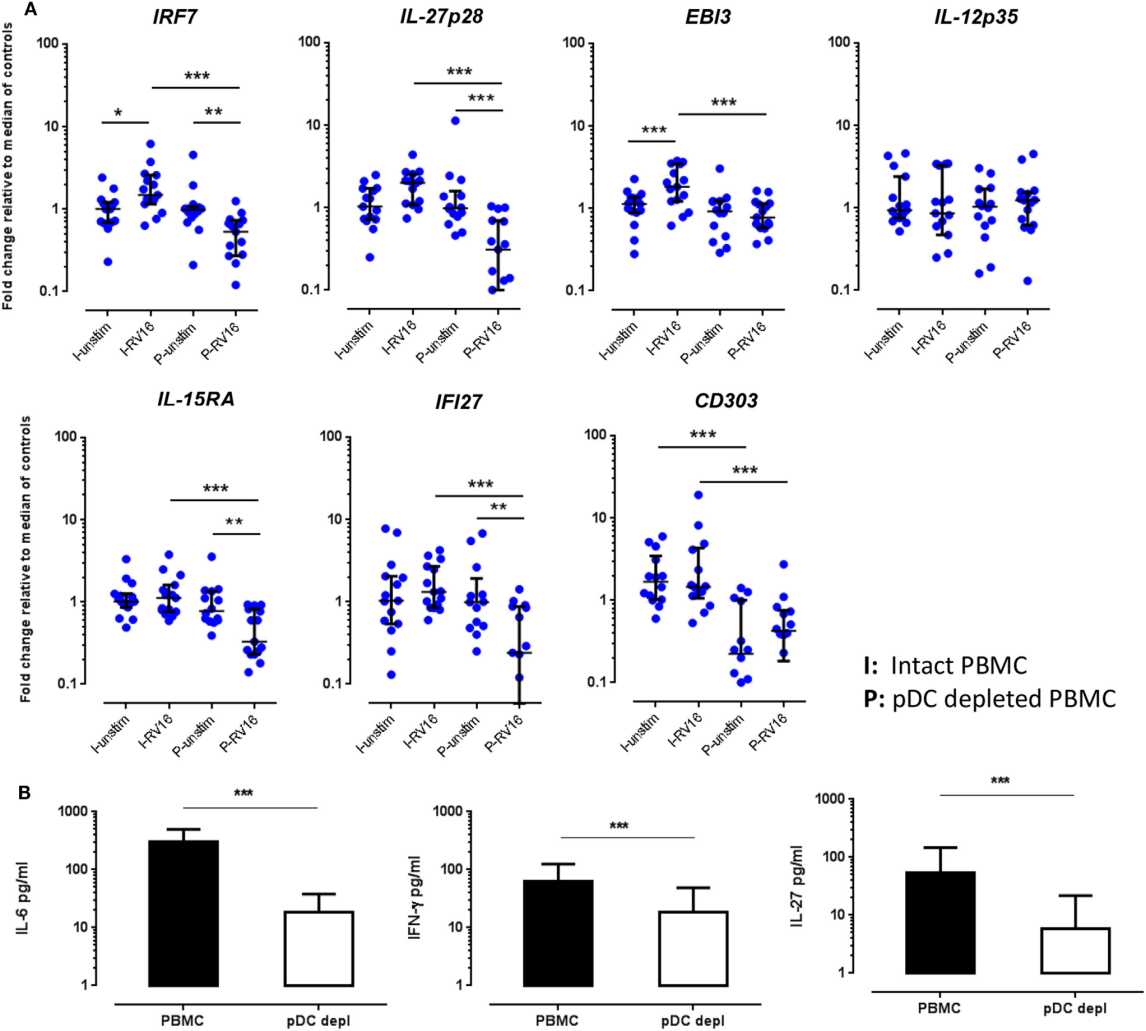
Validations experiments using real-time PCR (RT-PCR) and enzyme-linked immunosorbent assay (ELISA). Peripheral blood mononuclear cell (PBMC) and plasmacytoid dendritic cell (pDC)-depleted PBMC (*n* = 15) were cultured for 24 h in the absence or presence of human rhinoviruses strain 16 (RV16). mRNA expression of *IRF7, IL-27p28, EBI3, IL-12p35, IL-15RA, IFI27*, and *CD303* was determined by RT-PCR. Data represent fold change relative to median of controls against the average of two reference genes (*UBE2D2* and *B2M*) **(A)**. IL-6 and interferon (IFN)-γ were measured in culture supernatant by ELISA. Net cytokine values are shown (RV16-stimulated cultures minus the unstimulated cultures) **(B)**. All data represent mean ± SD. *p < 0.05, **p < 0.01, ***p < 0.001 (I, intact PBMC; P, pDC-depleted PBMC).

In the absence of virus exposure, intact PBMC and pDC-depleted PBMC expressed each of the aforementioned targets to a similar degree, with the exception of *CD303*, which was significantly lower in pDC-depleted PBMC compared to intact PBMC. Consistent with the microarray data, RT-PCR confirmed that *IRF7, IL-27p28, EBI3, IL-15RA*, and *IFI27* mRNA expression was significantly higher in intact PBMC than pDC-depleted PBMC following RV16 stimulation (Figure 5A). Note that RV16 induced higher *IRF7* and *EBI3* expression in the intact PBMC, but not in pDC-depleted PBMC. In fact, virus induced downregulation of several genes in the absence of pDC including *IRF7, IL-27, IL-15RA*, and *IFI27*. Expression of *IL-12p35* did not vary in relation to the presence or absence of RV16, or the presence or absence of pDC (Figure 5A). Similarly, in response to RV16 exposure, intact PBMC synthesized more IL-6, IFN-γ, and IL-27 protein than pDC-depleted PBMC (Figure 5B). Purified pDC produced more IFN-α and less IL-6 than intact PBMC (Figure S1 in Supplementary Material).

### Multiple Cytokines Can “Rescue” IFN-α Production in the Absence of pDC

**Figure 6:**
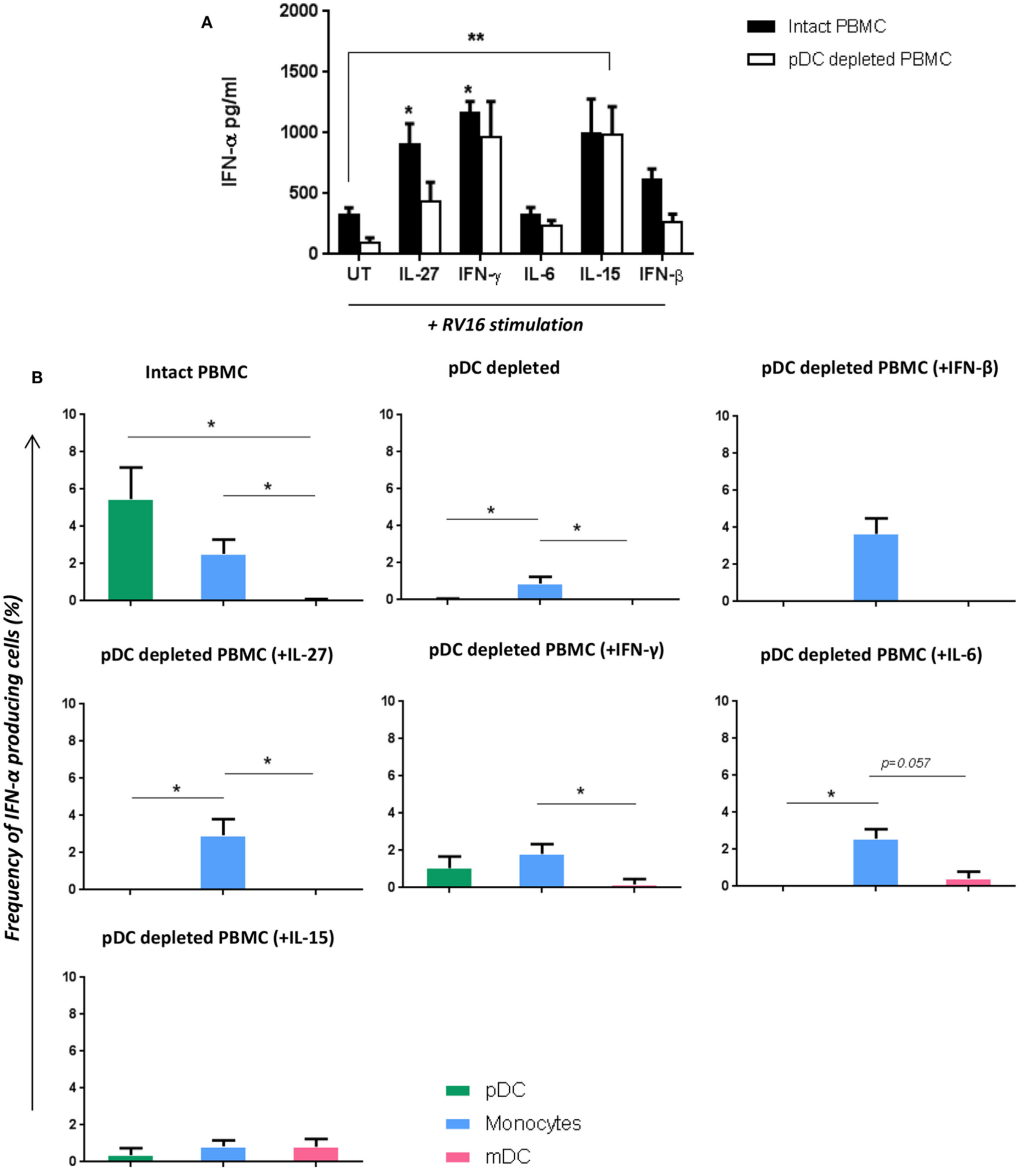
Addition of certain cytokines to plasmacytoid dendritic cell (pDC)-depleted cultures successfully rescues the interferon (IFN)-α response. Intact peripheral blood mononuclear cell (PBMC) (black bar) and pDC-depleted PBMC (white bar) were precultured with IL-27, IFN-γ, IL-6, IL-15, or IFN-β recombinant protein or with media only for 4 h at 37°C. All samples were subsequently stimulated with human rhinoviruses strain 16 (RV16) [multiplicity of infection (MOI) = 1] for a further 20 h. IFN-α protein was measured by ELISA (*p < 0.05; **p < 0.01; UT, untreated.) **(A)**, and IFN-α producing cell subsets were measured by Flow Cytometry (*p < 0.05) **(B)**. Data represent mean ± SD.

Having shown that multiple RV16 responsive cytokines are pDC dependent, we next sought to determine if adding back any of these missing cytokines would be sufficient to restore the IFN-α response and compensate for the absence of pDC. IFN-β was included as a positive control in these “rescue experiments” as it is known to induce IFN-α. Exogenous IL-15 and IFN-γ had the largest effects amongst all the cytokines tested, restoring IFN-α production, and even boosting it above that seen with RV16 exposed intact PBMC. The effect of exogenous IL-15 was statistically significant (*p* < 0.01), and while there was a trend for a significant effect of IFN-γ, this was not statistically significant. IL-27 and IL-6 were also able to rescue IFN-α release by virus exposed pDC-depleted cultures (Figure 6A). The addition of IL-15, IFN-γ (*p* < 0.05), or IL-27(*p* < 0.05) into the intact PBMC also enhance IFN-α release compared to the untreated RV16-stimulated PBMC.

With intact PBMC, pDC were the most frequent IFN-α producing cell, followed by monocytes, with mDC making a limited contribution. In the absence of pDC, IFN-α producing monocytes were even less frequent. However, supplementing pDC-depleted PBMC with IL-27, IFN-γ, IL-6, or IFN-β enabled monocyte to produce IFN-α. Addition of IL-15 to pDC cultures induced a small amount of IFN-α within mDC (CD1c^+^) (Figure 6B).

## Discussion

In this study, we used a combination of pDC depletion and gene expression profiling to further our understanding of the regulatory properties of pDC in the setting of HRV infection. As expected, pDC depletion led to a major reduction in HRV-induced IFN-α release, and this was associated with profound differences in gene expression between intact PBMC and pDC-depleted PBMC. Upstream regulator analysis suggested that pDC depletion resulted in deficient activation of pathways downstream of IRF7, IFN-γ, RICTOR, and TNF. Interrogation of the data with the Interferome database revealed that almost half of the HRV-induced transcriptome are known to be regulated by IFNs. Validation experiments using qPCR and/or ELISA confirmed many of the changes seen in the microarray, specifically the expression of *IRF7*, the two chains of IL-27 (*IL-*27*p28* and *EBI3*), *IL-15RA*, and *IFI27*, the synthesis of IL-6, IFN-γ and IL-27, and the extent to which these are highly pDC dependent. Finally, “rescue” experiments indicated that IFN-γ, IL-15, IL-27, and IL-6 are all able to compensate for a lack of pDC, restoring the IFN-α response. The extent to which IL-27 is pDC dependent was an important and unexpected finding to emerge from our study.

To our knowledge, our study is the first to show that HRV induces IL-27 expression and that this is pDC dependent. There is some evidence that IL-27 dysfunction contributes to asthma pathophysiology, though little attention has been directed to the role of IL-27 in HRV infections. In a murine asthma model, WSX-1 deficient mice (also known as IL-27Rα^−/−^ mice) exhibited enhanced lung pathology, characterized by goblet cell hyperplasia, infiltration of eosinophils, elevated serum IgE, and airway hyperresponsiveness (37). In humans, single nucleotide polymorphisms in IL-27p28 are associated with asthma susceptibility, increased IgE, and eosinophilia (38), while IL-27 expression may play a role in severe asthma (39). In the current study, we have shown that HRV induces marked IL-27 expression/production that is highly pDC dependent. It has recently been shown that IL-27 in combination with IFN-I and IFN-II can regulate type 2 innate lymphoid (ILC2) cells thereby restricting type 2 immunopathology (40). IL-27 and IFN-γ can also induce a T_reg_ population (T-bet^+^, CXCR3^+^) that produce higher IL-10 to limit T effector responses, thus reducing infection-induced pathology (41). Our previous studies have shown that pDC have the capacity to constrain type 2 immune responses to HRV (18, 42). The current study adds to these findings by showing that pDC depletion results in significant reduction of IFN-α, IFN-γ and IL-27 following HRV infection; all three of these cytokines are important regulators that might constrain type 2 response particularly in HRV infection. The extent to which pDC can enhance T_reg_ function and regulate ILC2 cells also warrants further investigation.

HRV induced expression of numerous genes, with profound differences in gene expression between intact PBMC and pDC-depleted PBMC. Many of the differentially expressed genes were associated with the induction of IFN expression, IFN signaling, and initiation of innate immunity. This suggests that pDC play a critical role in the host response to HRV. In the setting of pDC deficiency or pDC dysfunction, individuals are likely to be highly vulnerable to the deleterious effects of this usually innocuous virus. Relative deficiency of circulating pDCs during infancy has been shown to be a risk factor that predicts more frequent and more severe respiratory tract infections, wheezing, and a diagnosis of asthma in later childhood (1). Interestingly, we have shown that 70–80% of the HRV-responsive genes appear to be pDC dependent; however, Figure 4 indicates that only a proportion of these are IFN-I dependent. Based on the *Interferome V2.01* database, it appears that only a minority of the total HRV induced gene expressions are solely IFN-I dependent, with additional genes responsive to combinations of type II and type III IFNs. Notably, many of the pDC-dependent expressed genes appear to be IFN independent, highlighting the extent to which pDC regulate additional immune response genes *via* mechanisms that are independent of IFN-I signaling. A role for pDC in limiting immunopathology is well established (14) and there is emerging evidence that pDC modulate regulatory T-cell function (43).

Based on the upstream regulator analysis, we identified several HRV-induced molecular drivers that appear to be highly pDC dependent. Others have identified *IRF7* as a master regulator of IFN-I function that is highly expressed in pDC (16). Similarly, Bosco et al. have identified *IRF7* as a major “hub gene” expressed during asthma exacerbations (44) and have demonstrated that IRF7 regulates expression of multiple genes in HRV infected human epithelial cells. Not only does *IRF7* knockdown inhibit expression of genes involved in the antiviral response but also enhances expression of proinflammatory genes and oxidative stress response genes (45). Furthermore, in a murine model of HRV infection, inhibiting IRF7 limited neutrophil and macrophage influx of the lungs, while reducing IFN responses (46). Additional IFN-I-related genes are also important in establishing an antiviral state during infection. *IFI27* was an additional HRV-induced gene (47) that was identified in our experiments as being highly pDC dependent. IFI27 can directly inhibit replication and function of hepatitis C virus, another RNA virus (48) though whether IFI27 has similar effects on HRV is not known.

It is interesting that many of the HRV-responsive genes are not solely IFN-I regulated and require assistance from other types of IFN, particularly IFN-γ. At the 24 h time point, NK cells might be an important source of IFN-γ. Human and mouse models of experimental HRV infection have shown that activation of NK and CD8^+^ T cells requires virus induced expression of IL-15 and its receptor (IL-15Rα) in the nasal and lower airway mucosa (49). Moreover, in IL-15Rα^−/−^ mice, HRV infection results in a severely impaired IFN-γ expression, CD8^+^ T cell responses, and higher viral load in lung (49). In this study, we found that HRV stimulation also triggered enhanced IL-15 and IL-15Rα gene expression in human PBMC, but the expression of IL-15Rα was significantly lower in the absence of pDC. Therefore, we propose that low IFN-γ expression in the absence of pDC is likely secondary to deficient IFN-I-dependent IL-15Rα expression that can be partly overcome by provision of exogenous IL-15. In contrast, experimental HRV infections in mice indicate that IL-15 production can be induced independent of type I IFN, though the role of pDC was not examined in that study (49).

IL-6 is not only a general marker of inflammation but also contributes to inflammatory disease pathogenesis. In our study, HRV-induced IL-6 was also pDC dependent. Though pure pDC can produce IL-6, they are not the primary IL-6 producers, contributing with <15% of total production (Figure S1 in Supplementary Material). In allergic asthma, TLR7 activated pDC produce less IL-6, IFN-α, and TNF than pDC from healthy people (50). This is consistent with our finding that pDC depletion produced significantly lower IL-6 and IFN-α, and somewhat lower TNF-α, consistent with the notion that pDC function might be impaired in asthma. IL-6 signals *via* two types of receptors: membrane-bound IL-6 receptor (mIL-6R) and circulating soluble receptor (sIL-6R), and the use of these receptors can profoundly influence asthma pathogenesis (51). sIL6 has been shown to be induced by viral infection, and sIL-6R mediated antiviral activation *via* the p28 pathway and IFN-α could promote nuclear translocation of IFN regulatory factor 3 (IRF3) and NF-κB, thereby inducing activation of downstream IFN effector molecules such as 2′5′OAS, PKR, and Mx (52). It would be interesting for future studies to examine how pDC regulates sIL-6R, and better understand the relationship between sIL-6R, IL-27p28, and downstream IFN stimulatory genes production.

In this study, we have shown that multiple HRV responsive cytokines are pDC dependent, and supplementation with exogenous IL-27, IFN-γ, IL-6, or IL-15 can restore IFN-α response in HRV exposed, pDC-depleted culture. However, if pDC are the primary IFN-α producers (12), what is the mechanism responsible for this rescued IFN-α production in the absence of pDC? The ability of exogenous IL-27 to rescue IFN-α synthesis in pDC-depleted cultures could be mediated *via* the known ability of IL-27 to activate STAT-1 (39, 53). In addition, IL-27 can induce synthesis of the IFN inducible chemokine CXCL10 (IP-10) in human monocytes (53). As shown in Figure 6B, IL-27, IFN-γ, and IL-6 are all able to induce monocytes to produce IFN-α in the absence of pDC. In contrast, the ability of exogenous IL-15 to rescue IFN-α synthesis does not appear to involve monocytes, but a minor involvement of mDC was observed, so the source of IFN-α synthesis in this situation remains to be determined.

We employed a well-established method by our group to deplete pDC from PBMC *ex vivo*, confirming our previous findings that pDC are the principal IFN-α producer in HRV infection (12, 18). Flow cytometry and RT-PCR expression of CD303 confirmed the efficiency of pDC depletion in the current study. Importantly, HRV exposure *per se* did not modify CD303 expression. Nonetheless, we acknowledge there are a number of limitations of the study. Reagents for protein quantitation for some of the key genes were not commercially available so it was not possible to determine whether all changes in gene expression corresponded to changes in protein expression. The study used only a single strain of HRV (HRV-16), and examined a single time point. The HRV induced innate immune response is mediated by highly conserved pathogen-associated molecular patterns (PAMPs) such as ssRNA, and these are likely to be identical or very similar across multiple HRV strains. There might also be slightly difference between HRV major groups (90%) and HRV minor group (10%) due to the different receptors they use to gain entry to cells, and this needs to be addressed in further studies. Future studies using a wider range of HRV serotypes are necessary to better understand HRV induced adaptive immune response (e.g., antibody responses, T-cell responses), as these are likely to be more variable than innate immune responses. Finally, it is important to highlight the fact that pDC and other circulating immune cells do not usually encounter HRV in the circulation but rather in the airway mucosa, so it is important for future studies to examine interactions between pDC and airway epithelial cells, notwithstanding the technical challenges of such experiments. Nonetheless, our microarray findings provide a significant advance in current understanding of the immune response to HRV. The use of pDC depletion provides a powerful tool to understand the function of this rare but important leukocyte subset.

In conclusion, analysis of HRV activated gene expression patterns indicates the extent to which a small population of pDC are able to exert a profound effect on the immune response. pDC depletion led to major changes in upstream regulator, with 70–80% of the HRV activated genes appearing to be pDC dependent. Several pDC-dependent cytokines were identified, that when added to pDC-depleted cultures, were able to rescue the IFN-α response thereby providing important insight into the mechanisms by which pDC regulate immune responses to HRV. Better understanding of the role of pDC in regulating immune responses to HRV is likely to provide important insights into the reasons why this usually innocuous virus can have serious consequences in certain individuals, and why it is such a common inducer of worsening airway inflammation in those with asthma.

## Ethics Statement

The Metro South Human Research Ethics Committee approved the study, and all subjects provided written informed consent.

## Author Contributions

Conception and design: YX, AB, and JU; acquisition: YX, NT, DA, and OP; analysis and interpretation of data, revising the work for important intellectual content: YX, NT, DA, OP, JL, SP, AB, and JU; drafting the manuscript for important intellectual content: YX, NT, DA, and JU; and final approval of the version to be published: all authors.

## Conflict of Interest Statement

The authors declare that the research was conducted in the absence of any commercial or financial relationships that could be construed as a potential conflict of interest.

## Supplementary Material

Figure S1 The cytokine and expression level of genes of interested in pure pDC following RV16 stimulation. FACS purified pDC and PBMC (*n* = 2) were stimulated with RV16 for 24 h. IFN-α and IL-6 protein **(A,B)** in the cultured supernatant were measured using ELISA.

## References

[1] Upham JW Zhang G Rate A Yerkovich ST Kusel M Sly PD Plasmacytoid dendritic cells during infancy are inversely associated with childhood respiratory tract infections and wheezing. J Allergy Clin Immunol (2009) 124(4):707.e–13.e. 10.1016/j.jaci.2009.07.009 19733903

[2] Gern JE. The ABCs of rhinoviruses, wheezing, and asthma. J Virol (2010) 84(15):7418–26. 10.1128/jvi.02290-09 20375160 PMC2897627

[3] George SN Garcha DS Mackay AJ Patel AR Singh R Sapsford RJ Human rhinovirus infection during naturally occurring COPD exacerbations. Eur Respir J (2014) 44(1):87–96. 10.1183/09031936.00223113 24627537

[4] McNab F Mayer-Barber K Sher A Wack A O’Garra A. Type I interferons in infectious disease. Nat Rev Immunol (2015) 15(2):87–103. 10.1038/nri3787 25614319 PMC7162685

[5] Edwards MR Regamey N Vareille M Kieninger E Gupta A Shoemark A Impaired innate interferon induction in severe therapy resistant atopic asthmatic children. Mucosal Immunol (2013) 6(4):797–806. 10.1038/mi.2012.118 23212197 PMC3684776

[6] Wark PA Johnston SL Bucchieri F Powell R Puddicombe S Laza-Stanca V Asthmatic bronchial epithelial cells have a deficient innate immune response to infection with rhinovirus. J Exp Med (2005) 201(6):937–47. 10.1084/jem.20041901 15781584 PMC2213100

[7] Lewis TC Henderson TA Carpenter AR Ramirez IA McHenry CL Goldsmith AM Nasal cytokine responses to natural colds in asthmatic children. Clin Exp Allergy (2012) 42(12):1734–44. 10.1111/cea.12005 23181789 PMC4219353

[8] Proud D Turner RB Winther B Wiehler S Tiesman JP Reichling TD Gene expression profiles during in vivo human rhinovirus infection: insights into the host response. Am J Respir Crit Care Med (2008) 178(9):962–8. 10.1164/rccm.200805-670OC 18658112

[9] Subauste MC Jacoby DB Richards SM Proud D. Infection of a human respiratory epithelial cell line with rhinovirus. Induction of cytokine release and modulation of susceptibility to infection by cytokine exposure. J Clin Invest (1995) 96(1):549–57. 10.1172/jci118067 7615827 PMC185229

[10] Ghildyal R Dagher H Donninger H de Silva D Li X Freezer NJ Rhinovirus infects primary human airway fibroblasts and induces a neutrophil chemokine and a permeability factor. J Med Virol (2005) 75(4):608–15. 10.1002/jmv.20315 15714497

[11] Saba TG Chung Y Hong JY Sajjan US Bentley JK Hershenson MB. Rhinovirus-induced macrophage cytokine expression does not require endocytosis or replication. Am J Respir Cell Mol Biol (2014) 50(5):974–84. 10.1165/rcmb.2013-0354OC 24783958 PMC4068949

[12] Xi Y Finlayson A White OJ Carroll ML Upham JW Rhinovirus stimulated IFN-[alpha] production: how important are plasmacytoid DCs, monocytes and endosomal pH? Clin Trans Immunol (2015) 4(10):e46 10.1038/cti.2015.27 PMC4673444 26682054

[13] Reizis B Bunin A Ghosh HS Lewis KL Sisirak V. Plasmacytoid dendritic cells: recent progress and open questions. Annu Rev Immunol (2011) 29:163–83. 10.1146/annurev-immunol-031210-101345 21219184 PMC4160806

[14] Lynch JP Mazzone SB Rogers MJ Arikkatt JJ Loh Z Pritchard AL The plasmacytoid dendritic cell: at the cross-roads in asthma. Eur Respir J (2014) 43(1):264–75. 10.1183/09031936.00203412 23429916

[15] Siegal FP Kadowaki N Shodell M Fitzgerald-Bocarsly PA Shah K Ho S The nature of the principal type 1 interferon-producing cells in human blood. Science (1999) 284(5421):1835–7. 10.1126/science.284.5421.1835 10364556

[16] Honda K Yanai H Negishi H Asagiri M Sato M Mizutani T IRF-7 is the master regulator of type-I interferon-dependent immune responses. Nature (2005) 434(7034):772–7. 10.1038/nature03464 15800576

[17] Swiecki M Gilfillan S Vermi W Wang Y Colonna M. Plasmacytoid dendritic cell ablation impacts early interferon responses and antiviral NK and CD8(+) T cell accrual. Immunity (2010) 33(6):955–66. 10.1016/j.immuni.2010.11.020 21130004 PMC3588567

[18] Pritchard AL Carroll ML Burel JG White OJ Phipps S Upham JW Innate IFNs and plasmacytoid dendritic cells constrain Th2 cytokine responses to rhinovirus: a regulatory mechanism with relevance to asthma. J Immunol (2012) 188(12):5898–905. 10.4049/jimmunol.1103507 22611238

[19] Sanders SP Siekierski ES Porter JD Richards SM Proud D. Nitric oxide inhibits rhinovirus-induced cytokine production and viral replication in a human respiratory epithelial cell line. J Virol (1998) 72(2):934–42. 9444985 10.1128/jvi.72.2.934-942.1998 PMC124563

[20] Roponen M Yerkovich ST Hollams E Sly PD Holt PG Upham JW. Toll-like receptor 7 function is reduced in adolescents with asthma. Eur Respir J (2010) 35(1):64–71. 10.1183/09031936.00172008 19643938

[21] Core Team R R: A Language and Environment for Statistical Computing. Vienna, Austria: R Foundation for Statistical Computing (2016).

[22] Du P Kibbe WA Lin SM. Lumi: a pipeline for processing Illumina microarray. Bioinformatics (2008) 24(13):1547–8. 10.1093/bioinformatics/btn224 18467348

[23] Ritchie ME Phipson B Wu D Hu Y Law CW Shi W Limma powers differential expression analyses for RNA-sequencing and microarray studies. Nucleic Acids Res (2015) 43(7):e47. 10.1093/nar/gkv007 25605792 PMC4402510

[24] Shi W Oshlack A Smyth GK. Optimizing the noise versus bias trade-off for Illumina whole genome expression BeadChips. Nucleic Acids Res (2010) 38(22):e204. 10.1093/nar/gkq871 20929874 PMC3001098

[25] Barbosa-Morais NL Dunning MJ Samarajiwa SA Darot JF Ritchie ME Lynch AG A re-annotation pipeline for Illumina BeadArrays: improving the interpretation of gene expression data. Nucleic Acids Res (2010) 38(3):e17. 10.1093/nar/gkp942 19923232 PMC2817484

[26] Dunning M Lynch A Eldridge M IlluminaHumanv4.db: Illumina HumanHT12v4 Annotation Data (Chip IlluminaHumanv4). R package version 1.26.0 ed. (2015).

[27] Ritchie ME Diyagama D Neilson J van Laar R Dobrovic A Holloway A Empirical array quality weights in the analysis of microarray data. BMC Bioinformatics (2006) 7:261. 10.1186/1471-2105-7-261 16712727 PMC1564422

[28] Smyth GK Michaud J Scott HS. Use of within-array replicate spots for assessing differential expression in microarray experiments. Bioinformatics (2005) 21(9):2067–75. 10.1093/bioinformatics/bti270 15657102

[29] Benjamini Y Hochberg Y Controlling the false discovery rate: a practical and powerful approach to multiple testing. J R Stat Soc Ser B (1995) 57(1):289–300.

[30] Adler D Murdoch D Others rgl: 3D Visualization Using OpenGL. R package version 0.96.0 ed. (2016).

[31] Warnes G Bolker B Bonebakker L Gentleman R Liaw W Lumley T gplots: Various R Programming Tools for Plotting Data. R package version 3.0.1 ed. (2016).

[32] Kramer A Green J Pollard J Jr Tugendreich S. Causal analysis approaches in ingenuity pathway analysis. Bioinformatics (2014) 30(4):523–30. 10.1093/bioinformatics/btt703 24336805 PMC3928520

[33] Troy NM Hollams EM Holt PG Bosco A. Differential gene network analysis for the identification of asthma-associated therapeutic targets in allergen-specific T-helper memory responses. BMC Med Genomics (2016) 9:9. 10.1186/s12920-016-0171-z 26922672 PMC4769846

[34] Pritchard AL White OJ Burel JG Carroll ML Phipps S Upham JW. Asthma is associated with multiple alterations in anti-viral innate signalling pathways. PLoS One (2014) 9(9):e106501. 10.1371/journal.pone.0106501 25203745 PMC4159236

[35] Rusinova I Forster S Yu S Kannan A Masse M Cumming H Interferome v2.0: an updated database of annotated interferon-regulated genes. Nucleic Acids Res (2013) 41(Database issue):D1040–6. 10.1093/nar/gks1215 23203888 PMC3531205

[36] Yoshida H Hunter CA. The immunobiology of interleukin-27. Annu Rev Immunol (2015) 33:417–43. 10.1146/annurev-immunol-032414-112134 25861977

[37] Miyazaki Y Inoue H Matsumura M Matsumoto K Nakano T Tsuda M Exacerbation of experimental allergic asthma by augmented Th2 responses in WSX-1-deficient mice. J Immunol (2005) 175(4):2401–7. 10.4049/jimmunol.175.4.2401 16081811

[38] Chae SC Li CS Kim KM Yang JY Zhang Q Lee YC Identification of polymorphisms in human interleukin-27 and their association with asthma in a Korean population. J Hum Genet (2007) 52(4):355–61. 10.1007/s10038-007-0123-8 17318299

[39] Xie M Mustovich AT Jiang Y Trudeau JB Ray A Ray P IL-27 and type 2 immunity in asthmatic patients: association with severity, CXCL9, and signal transducer and activator of transcription signaling. J Allergy Clin Immunol (2015) 135(2):386–94. 10.1016/j.jaci.2014.08.023 25312760 PMC4324337

[40] Duerr CU McCarthy CD Mindt BC Rubio M Meli AP Pothlichet J Type I interferon restricts type 2 immunopathology through the regulation of group 2 innate lymphoid cells. Nat Immunol (2016) 17(1):65–75. 10.1038/ni.3308 26595887 PMC9135352

[41] Hall AO Beiting DP Tato C John B Oldenhove G Lombana CG The cytokines interleukin 27 and interferon-gamma promote distinct Treg cell populations required to limit infection-induced pathology. Immunity (2012) 37(3):511–23. 10.1016/j.immuni.2012.06.014 22981537 PMC3477519

[42] Pritchard AL White OJ Burel JG Upham JW. Innate interferons inhibit allergen and microbial specific T(H)2 responses. Immunol Cell Biol (2012) 90(10):974–7. 10.1038/icb.2012.39 22825591

[43] Baeyens A Saadoun D Billiard F Rouers A Gregoire S Zaragoza B Effector T cells boost regulatory T cell expansion by IL-2, TNF, OX40, and plasmacytoid dendritic cells depending on the immune context. J Immunol (2015) 194(3):999–1010. 10.4049/jimmunol.1400504 25548233

[44] Bosco A Ehteshami S Panyala S Martinez FD. Interferon regulatory factor 7 is a major hub connecting interferon-mediated responses in virus-induced asthma exacerbations in vivo. J Allergy Clin Immunol (2012) 129(1):88–94. 10.1016/j.jaci.2011.10.038 22112518 PMC3246116

[45] Bosco A Wiehler S Proud D. Interferon regulatory factor 7 regulates airway epithelial cell responses to human rhinovirus infection. BMC Genomics (2016) 17:76. 10.1186/s12864-016-2405-z 26810609 PMC4727386

[46] Girkin J Hatchwell L Foster P Johnston SL Bartlett N Collison A CCL7 and IRF-7 mediate hallmark inflammatory and IFN responses following rhinovirus 1B infection. J Immunol (2015) 194(10):4924–30. 10.4049/jimmunol.1401362 25847975 PMC4417644

[47] Heinonen S Jartti T Garcia C Oliva S Smitherman C Anguiano E Rhinovirus detection in symptomatic and asymptomatic children: value of host transcriptome analysis. Am J Respir Crit Care Med (2016) 193(7):772–82. 10.1164/rccm.201504-0749OC 26571305 PMC4824929

[48] Itsui Y Sakamoto N Kakinuma S Nakagawa M Sekine-Osajima Y Tasaka-Fujita M Antiviral effects of the interferon-induced protein guanylate binding protein 1 and its interaction with the hepatitis C virus NS5B protein. Hepatology (2009) 50(6):1727–37. 10.1002/hep.23195 19821486

[49] Jayaraman A Jackson DJ Message SD Pearson RM Aniscenko J Caramori G IL-15 complexes induce NK- and T-cell responses independent of type I IFN signaling during rhinovirus infection. Mucosal Immunol (2014) 7(5):1151–64. 10.1038/mi.2014.2 24472849 PMC4284198

[50] Bratke K Prieschenk C Garbe K Kuepper M Lommatzsch M Virchow JC. Plasmacytoid dendritic cells in allergic asthma and the role of inhaled corticosteroid treatment. Clin Exp Allergy (2013) 43(3):312–21. 10.1111/cea.12064 23414539

[51] Ullah MA Revez JA Loh Z Simpson J Zhang V Bain L Allergen-induced IL-6 trans-signaling activates gammadelta T cells to promote type 2 and type 17 airway inflammation. J Allergy Clin Immunol (2015) 136(4):1065–73. 10.1016/j.jaci.2015.02.032 25930193

[52] Wang Q Chen X Feng J Cao Y Song Y Wang H Soluble interleukin-6 receptor-mediated innate immune response to DNA and RNA viruses. J Virol (2013) 87(20):11244–54. 10.1128/jvi.01248-13 23946454 PMC3807281

[53] Guzzo C Che Mat NF Gee K. Interleukin-27 induces a STAT1/3- and NF-kappaB-dependent proinflammatory cytokine profile in human monocytes. J Biol Chem (2010) 285(32):24404–11. 10.1074/jbc.M110.112599 20519510 PMC2915676

