# Supplementary figures and images for: Critical Role of Plasmacytoid Dendritic Cells in Regulating Gene Expression and Innate Immune Responses to Human Rhinovirus-16
Source: Front Immunol. 2017 Oct 25;8:1351. doi: 10.3389/fimmu.2017.01351 (PMC5660993; doi:10.3389/fimmu.2017.01351)

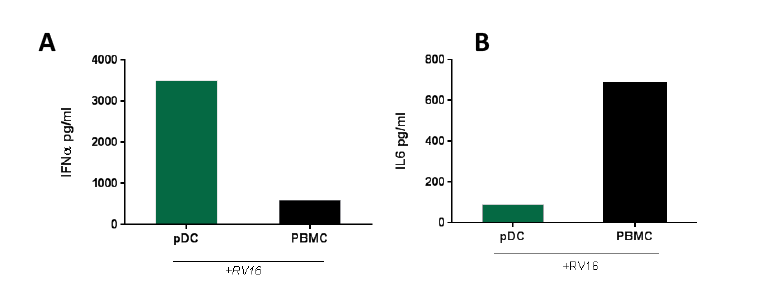

Supplement: Figure S1 — The cytokine and expression level of genes of interested in pure pDC following RV16 stimulation. FACS purified pDC and PBMC (n = 2) were stimulated with RV16 for 24 h. IFN-α and IL-6 protein (A,B) in the cultured supernatant were measured using ELISA. [file Image_1.tif]
