# Supplementary material for: Critical Role of Plasmacytoid Dendritic Cells in Regulating Gene Expression and Innate Immune Responses to Human Rhinovirus-16
Source: Front Immunol. 2017 Oct 25;8:1351. doi: 10.3389/fimmu.2017.01351 (PMC5660993; doi:10.3389/fimmu.2017.01351)
Supplement: Supplementary file 2 [file Table_1.docx]

| **Oligo Name:**  **Supplemental Table- S1** | **Forward primer (5'-3')** | **Reverse primer (5'-3')** |
| --- | --- | --- |
| ***CD303*** | *GGC AGT CGT ATC CAT CTT GCT C* | *ATG ACG CAG GTC AGG CTT GGA T* |
| ***IRF-7*** | *GGC TGG AAA ACC AAC TTC C* | *GCC TCT GCC TCA GTC TGG T* |
| ***IL-27p28*** | *GGA ATC TCA CCT GCC AGG AGT G* | *TGG TGG AGA TGA AGC AGA GAC G* |
| ***EBI3*** | *CTG GAT CCG TTA CAA GCG TCA G* | *CAC TTG GAC GTA GTA CCT GGC T* |
| ***IL-15RA*** | *TGG CTA TCT CCA CGT CCA CTG T* | *CAT GGC TTC CAT TTC AAC GCT GG* |
| ***IFI27*** | *CGT CCT CCA TAG CAG CCA AGA T* | *ACC CAA TGG AGC CCA GGA TGA A* |
| ***IL-12p35 (IL-12A)*** | *GAT GAG CTG ATG CAG GCC* | *AGT CCT CCA CCT CGT TGT CCG TGA* |
| ***UBE2D2*** | *ATG GCA GCA TTT GTC TTG ATA TTC TAC* | *TGG ATT GGG ATC ACA CAA CAG A* |
| ***B2M*** | *AGG CTA TCC AGC GTA CTC CAA AGA* | *CGG ATG GAT GAA ACC CAG ACA CAT* |
