# Supplementary material for: Critical Role of Plasmacytoid Dendritic Cells in Regulating Gene Expression and Innate Immune Responses to Human Rhinovirus-16
Source: Front Immunol. 2017 Oct 25;8:1351. doi: 10.3389/fimmu.2017.01351 (PMC5660993; doi:10.3389/fimmu.2017.01351)
Supplement: Supplementary file 3 [file Table_2.PDF]

SUPPLEMENTAL TABLE-S2

(A) pDC depleted PBMC vs intact PBMC differential response to RV16

Symbol	Name	LogFC	P. Value	Adj.p.Val
IRF7	interferon regulatory factor 7	-1.4455	3.23E-07	1.29E-05
IFN-γ	interferon, gamma	-1.5165	0.00042	0.0048
IL-15RA	interleukin 15 receptor, alpha	-1.4077	5.83E-15	3.96E-12
IL-6	interleukin 6 (interferon, beta 2)	-2.1793	5.84E-15	3.96E-12
IL-27	interleukin 27	-1.7909	5.35E-18	4.23E-14
IFI-27	interferon, alpha-inducible protein 27	-3.110	1.70E-07	7.45E-06

(B) Upstream Regulator analysis (pDC depleted PBMC +RV16 VS PBMC + RV16)

Names of genes	Molecule Type	Predicted activation state	Activation Z-score	P-Value of overlap
IRF7	Transcription regulator	inhibited	-8.051	1.44E-43
IFN-γ	Cytokine	inhibited	-8.308	1.46E-40
IL-15	Cytokine	Inhibited	-2.264	3.25E-14
IL-6	Cytokine	Inhibited	-2.293	1.22E-10
IL-27p28	Cytokine	inhibited	-2.425	3.98E-7
